# Interplay between Type 2 Transglutaminase (TG2), Gliadin Peptide 31-43 and Anti-TG2 Antibodies in Celiac Disease

**DOI:** 10.3390/ijms21103673

**Published:** 2020-05-23

**Authors:** Stefania Martucciello, Silvia Sposito, Carla Esposito, Gaetana Paolella, Ivana Caputo

**Affiliations:** 1 Department of Chemistry and Biology, University of Salerno, 84084 Fisciano (SA), Italy; smartucciello@unisa.it (S.M.); cesposito@unisa.it (C.E.); gpaolella@unisa.it (G.P.); 2 European Laboratory for the Investigation of Food-Induced Diseases (ELFID), University of Salerno, 84084 Fisciano (SA), Italy; ssposito@unisa.it

**Keywords:** type 2 transglutaminase, celiac disease, anti-TG2 antibodies, gliadin peptide31-43

## Abstract

Celiac disease (CD) is a common intestinal inflammatory disease involving both a genetic background and environmental triggers. The ingestion of gluten, a proteic component of several cereals, represents the main hexogen factor implied in CD onset that involves concomitant innate and adaptive immune responses to gluten. Immunogenicity of some gluten sequences are strongly enhanced as the consequence of the deamidation of specific glutamine residues by type 2 transglutaminase (TG2), a ubiquitous enzyme whose expression is up-regulated in the intestine of CD patients. A short gluten sequence resistant to intestinal proteases, the α-gliadin peptide 31-43, seems to modulate TG2 function in the gut; on the other hand, the enzyme can affect the biological activity of this peptide. In addition, an intense auto-immune response towards TG2 is a hallmark of CD. Auto-antibodies exert a range of biological effects on several cells, effects that in part overlap with those induced by peptide 31-43. In this review, we delineate a scenario in which TG2, anti-TG2 antibodies and peptide 31-43 closely relate to each other, thus synergistically participating in CD starting and progression.

## 1. Introduction

The enzyme type 2 transglutaminase (TG2) plays a key role in the pathogenesis of celiac disease (CD), primarily for its enzymatic activity that transforms common food proteins, i.e., gluten proteins contained in cereals, in unhealthy molecules for genetic predisposed individuals [1]. However, a class of gluten peptides, in particular peptide 31-43 of the α-gliadin (P31-43), does not require TG2-induced modifications to be “toxic” for the organism [2]. P31-43 exerts damaging effects directly on cells with which it comes in contact [2]. Interestingly, it is able to modulate TG2 activity and expression; in turn, TG2 may regulate some effects induced by P31-43. Auto-antibodies against TG2, abundantly produced at an early stage of CD development, have themselves a biological activity when interacting with TG2 on the cell surface and in the extra-cellular matrix (ECM) [3]. In some cases, they are able to modulate effects produced by P31-43 stimulation [3]. In this review, we have examined known or potential relationships between TG2, P31-43 and antibodies to TG2, trying to highlight the thin thread connecting them in the molecular mechanism of CD pathogenesis.

## 2. Generality on Celiac Disease 

Celiac disease (CD) is a complex inflammatory and auto-immune disorder triggered by the ingestion of gluten, a proteic component of several cereals, such as wheat, barley and rice [4]. Gluten proteins, at the level of the intestinal mucosa, cause an immune response that leads to an extensive mucosal remodeling and organ damage [4]. CD patients can present a certain grade of mucosal atrophy and crypt hyperplasia, including an increase of the intra-epithelial lymphocytes infiltrate [4]. Besides classical intestinal manifestations of the disease (diarrhea, malabsorption, anemia, weight loss, growth delay, etc.), there is a wide range of possible extra-intestinal symptoms including bone, liver, skin and neurological manifestations [4,5]. An important hallmark of CD is the presence of an auto-immune response towards one main auto-antigen represented by the enzyme TG2 [6]. The research in sera of antibodies to TG2, in particular of IgA class, represents the first screening level for clinical diagnosis of active CD [7,8]. Antibodies to other members of transglutaminase (TG) family can be sometimes detected; for example, antibodies to epidermal (type 3) TG are a typical marker of dermatitis herpetiformis, the dermal manifestation of CD [9,10], whereas antibodies to neuronal (type 6) TG form neuronal deposits in patients affected by gluten ataxia [11]. Even if gluten is the main environmental trigger for CD, other concomitant factors can contribute to the disease, for example, viral infections or microbiota alterations [12,13,14]. However, the environmental contribution is not sufficient to trigger CD, and a genetic background is also necessary. It consists of the presence of particular haplotypes of the human leukocytes antigen (HLA) system of class II, codifying molecules that bind antigens on antigen presenting cells (APC) [4]. CD patients almost invariably possess HLA-DQ2 variants (in more that 90% of subjects) or HLA-DQ8 variants [15]. However, accordingly to the latest CD prevalence studies, these haplotypes are present in about 40%–50% of the population [16], whereas the incidence of CD is estimated to be about 1% in the general population [17]. Currently, genetic tests targeted to find HLA-DQ2/8 haplotypes have only a negative predictive value in the diagnostic practice. A lot of additional non-HLA genes are under investigation to establish their contribution to the CD genetic susceptibility and data overall come from studies of genome-wide association [18]. 

## 3. Gluten Proteins and the Adaptive/Innate Immune Response

Gluten is a heterogeneous mixture of seed-storage proteins present in cereals such as wheat, barley, rye and oats [19]. From a biochemical point of view, gluten is composed by prolamines, i.e., proteins very rich in repetitive sequences containing glutamine and proline residues. Prolamines from wheat divide into two groups named gliadins (subdivided in α/β, γ and ω types), the alcohol-soluble fraction, and glutenins (subdivided in low molecular and high molecular weight subunits), the alcohol-insoluble fraction. Both gliadins and glutenins are toxic for CD patients, however gliadin sequences are better characterized in the context of CD onset [20,21]. The molecular basis of gluten toxicity is related to its typical aminoacidic composition. In gliadins, the high proline content (15%-20%) renders gliadin polypeptides only partially hydrolyzed by digestive peptidases, thus they may reach a high concentration at the level of gut lumen and epithelium. In addition, the very high glutamine content (35%-38%) is crucial for gliadin immunogenic properties. A lot of sequences containing short repeated motifs have been characterized that are recognized by HLA-DQ2/8 on APC and presented to the immune system [20,21]. However, immunogenicity of gliadin peptides is greatly enhanced by an enzymatic modification catalyzed by TG2 at the level of the intestinal mucosa. TG2 deamidates some specific glutamine residues in the gliadin sequence, producing glutamic acid residues, negatively charged, better fitting into the groove of HLA-DQ2/8 recognizing site [1]. The most immunogenic gliadin sequence characterized until now is the α2-gliadin 33-mer (sequence 57–89), containing six overlapping copies of three different DQ2-restricted epitopes, including the sequence PQLP [20,22]. Presentation of deamidated immunogenic gliadin peptides by APC to CD4+ T cells elicits an adaptive immune response, with the release of pro-inflammatory cytokines (mainly interferon (INF)-γ); the TH1-mediated response is accompanied by an innate immune response involving upregulation of interleukin 15 (IL15) expression and CD8+ T cells recruitment; combined responses finally cause an extensive damage to the intestinal mucosa [4,23]. Immunogenicity of gliadin is not the only pathogenetic feature of gliadin peptides. Other large indigested polypeptides can accumulate in the intestinal lumen and display the ability to trigger an innate immune and various cell stress responses [2,23]. This is the case of the 25-mer (sequence 31-55 of α-gliadin) and of the shorter P31-43 [24,25]. At present, the only effective therapy for CD is represented by a strict and long-life gluten-free diet [26].

## 4. The Multi-Functionality of TG2

The enzymatic deamidation, which is of crucial importance in enhancing gliadin immunogenicity, is only one of several catalytic activities displayed by TG2. This protein belongs to a wide family of cross-linking enzymes with members in all reigns of the living [27]. Well-known examples of this family are the microbial TG, largely employed in several industrial applications [28], and Factor XIIIa, a zymogen implicated in the final steps of blood coagulation, where it stabilizes the fibrin clot [29]. TG2, also defined “tissue” TG, is ubiquitously present in almost all tissues and organs in mammals and its expression is finely tuned by hypoxic and inflammatory molecules [30]. TG2 shows a particular wide distribution into the cell: it is mainly a cytosolic protein, but it is also present in the nucleus, mitochondria and in association with the plasmatic membrane (both at the inner and at the outer face) [27,31]. Through an unconventional secretion mechanism, TG2 reaches the cell surface in association with integrins and can be released into ECM [32]. A lot of functions have been attributed to TG2, depending on its main catalytic activity, consisting of the formation, in a Ca^2+^-dependent manner, of an isopeptide bond between lateral chains of glutamine and lysine residues present into proteins. Due to this transamidating activity, TG2 acts as a stabilizing enzyme of protein networks, such as those formed by ECM proteins [30,31]. In the cytosol, TG2 cross-linking activity may regulate inflammatory responses and autophagy; for example, TG2 activity leads to the formation of polymers of IkBα and its consequential proteasomal degradation, causing Nf_K_B activation; TG2 may also cross-link beclin-1, thus inhibiting the autophagic process [30]. In the nucleus, TG2 transamidates histones and some transcriptional factors, thus regulating gene expression [30]. When available, a polyamine, instead of a lysine, can be linked to a glutamine residue; polyamination seems to contribute to modulate the function of TG2 substrates [33]. In the absence of amines and at a slightly acidic pH, TG2 can deamidate specific glutamine residues, as occurs for glutamines in gliadin [34]. All these enzymatic activities (transamidation, polyamination and deamidation) are strictly regulated by the availability of Ca^2+^ ions. TG2 is inactive at the very low Ca^2+^ concentration in the cytosol, but is rapidly activated after perturbation of Ca^2+^ homeostasis. Actually, the modulation of TG2 activity is very complex and involves conformational changes between an open active conformation, bound to Ca^2+^, and a closed inactive conformation, bound to GTP, a negative regulator of the transamidating activity [35,36]. Moreover, TG2 activity is also regulated by factors that modulate its redox state, such as thioredoxin-1 and the endoplasmic reticulum (ER)-resident protein p57 [37,38]. TG2 is considered a multifunctional protein, as it displays other less characterized catalytic properties, such as a GTPase activity, by which it participates in transducing signals at the inner side of plasmatic membrane [39], a protein disulfide isomerase activity, by which it contributes to mitochondrial function [40], and an ATP-dependent kinase activity, by which it may exert a transcriptional regulation [31]. Finally, TG2 can function as an adapter/scaffolding/signaling protein, independently from its enzymatic activities [31]. In particular, in the nucleus, TG2 may act as a transcriptional co-regulator in a catalytic independent manner [30]. In addition, at the level of cell surface, TG2 participates in the out-in signaling by interacting not only with integrins but also with some growth factor receptors [31]. It is evident that the framework of TG2 functions appears very complex and a lot of physiological processes, including matrix remodeling, cell death, differentiation, migration, etc., see TG2 as a key regulator. Consequently, a defective function of TG2 can contribute to the pathogenesis of certain cancers, of neurodegenerative disorders, of fibrotic states and autoimmune disease, including CD [1,41,42].

## 5. TG2 in the Intestine and the Auto-immune Response

It has been reported that TG2 expression is increased in the inflamed intestinal mucosa of CD patients [43,44,45]. It accumulates at the extracellular level, in lamina propria, and at the level of enterocytes. A general activation of TG2 catalytic activity has also been hypothesized in inflamed sites with potential negative consequence on immunoreactive response to gliadin peptides through the process of deamidation [43,46]. Moreover, TG2 seems to be responsible of the auto-immune response in CD. TG2 may form covalent complexes between itself (due to the presence of auto-reactive lysine residues) and gliadin peptides (due to abundant glutamine residues). This reaction is at the basis of a hapten-carrier-like model for the production of auto-antibodies to TG2 [47]. According to this model, complexes between TG2 and gliadin can be recognized and processed by TG2-specific B-cells (HLA-DQ2/8 positive) which, acting as APC, can present gliadin fragments to CD4+ T cells, activating them. These T cells can, in turn, stimulate B-cells and promote the auto-reactive immune response. Alternative models that explain the generation of auto-antibodies have been proposed, including the mechanism of molecular mimicry involving viral proteins [48] or the mechanism of production of neopitopes involving TG2 [1,49]. The hypothesis that the auto-immune response depend on gliadin-specific CD4+ T cells is supported by the fact that anti-TG2 antibodies gradually disappear after removing gluten from the diet [50]. Interestingly, the production and the deposition of anti-TG2 antibodies in the lamina propria is a very early event in CD and precedes the mucosal damage [51]. After their production, antibodies can be released in the gut lumen and in blood circulation, thus reaching and accumulating in other organs. The presence of anti-TG2 antibodies of IgA class, not only in sera but also in the intestinal mucosa, is considered a specific and sensible diagnostic marker of CD. Moreover, in association with a normal or slightly inflamed duodenal mucosa and in the absence of symptoms, the presence of anti-TG2 antibodies in sera is considered a condition of “potential” CD [52]. In potential CD patients, intestinal deposits of IgA anti-TG2 may be present and seem to be associated with the risk of developing villous atrophy [53]. However, the intestinal production of anti-TG2 antibodies seems not to have an absolute specificity for CD, but could be a prerogative of an inflammatory state at the intestinal level [54]. Finally, in the intestine of subjects with selective IgA deficiency, a condition frequently associated with CD, the deposition of compensatory anti-TG2 antibodies of IgM class has been found [55].

### Biological Activities of Anti-TG2 Antibodies

An increasing interest on possible biological functions of auto-antibodies to TG2 into the organism has been registered in the last years. Since antibodies are not able to over cross the plasmatic membrane, they have to act on the cell surface, where they may interact with membrane-bound TG2 and eventually they can be internalized together with TG2, during the normal turnover of this protein. Studies on the ability of anti-TG2 antibodies to modulate TG2 transamidating activity have given quite contrasting results, thus it remains unclear whether auto-antibodies interfere in vivo with the repairing function of TG2 in ECM or may block those reactions necessary to the breakdown of tolerance to gluten (i.e., deamidation and transamidation involving gluten peptides) [3]. In an enterocyte-like cell line (Caco-2), anti-TG2 antibodies, by interacting with the cell-surface TG2, act as signaling molecules able to induce proliferation, actin reorganization and Ca^2+^ mobilization [56,57]. In an endothelial cell model, celiac antibodies significantly modify the expression profile of genes involved in angiogenesis regulation and induce several defects in cell adhesion and polarization [58,59]. In a mouse model, the intraperitoneal injection of anti-TG2 antibodies causes an alteration in the smallintestinal mucosal morphology and an increased cellular infiltration in the lamina propria [60]. The general idea that emerges from all these studies, performed in in vitro and in vivo models, is that antibodies to TG2 could have a role in CD pathogenesis as they are able to reproduce several features of CD intestinal mucosa, such as enhanced proliferation and reduced differentiation, altered permeability, cell architecture modification, etc. [3]. It is also conceivable that, in the frequent cases of IgA immunodeficiency, IgM deposits against anti-TG2 in the gut could compensate the absence of IgA anti-TG2, thus similarly contributing to the mucosal remodeling. Finally, recent work focused the attention on an idiotype/anti-idiotype network in CD that could play a role in the very early stage of disease [61]. Authors described the presence of a detectable humoral response to anti-TG2 antibodies in patients without mucosal lesions or, to a lesser extent, with only the genetic predisposition to CD, which would have an immunoregulatory protective role in CD onset. They hypothesized that high levels of anti-idiotype antibodies (i.e., antibodies to anti-TG2 antibodies) in sera of genetically predisposed individuals (but absent in full-blown CD patients) could postpone, or even block, the potential dangerousness of idiotypes (i.e., antibodies to TG2). 

## 6. The Gliadin Peptide 31-43 (P31-43)

Among peptides obtained by the in vitro digestion in a multi-compartment system, P31-43 and the longer peptide 31-49 have been detected [25]. Thus, it is reasonable that P31-43 comes in contact with enterocytes of the gut epithelium. After this contact, P31-43 can have different fates. First, it can cross the intestinal barrier through a paracellular route [17]. An increased mucosal permeabilization seems just favored by P31-43 [62,63], thus the peptide enhances its own penetrative ability. Second, P31-43 can be internalized by enterocytes and partially concentrate in the endosomal compartment, or can be released in the lamina propria [64]. Recently, a great interest has been noticed on several aspects regarding P31-43 in the context of CD, from its structural features, to the way it interacts with cells, to its biological effects in the cell and biopsy culture models.

### 6.1. Structural Properties of P31-43

From biophysical studies investigating the secondary structure of P31-43 emerges that this peptide is able to self-organize in solution as a polyproline II conformation, in equilibrium with β-sheets-like structures [65,66]. Another study highlights the existence of two conformational structures around the amide bond of the two adjacent prolines 38 and 39 [67]. P31-43 is also able to self-assembly into oligomers in the multinanometer scale [65]. Moreover, peptide concentration greatly influences size and complexity of oligomers and from this finding it has been suggested that P31-43 oligomers may play a role in modulating the non-HLA mediated effects of gliadin, such as cellular stress and NLRP3 inflammasome activation [66]. Oligomers may also work as a reserve that protect P31-43 from degradation, and, as a consequence, amplify toxic potentiality of the peptide [68]. Finally, the three-dimensional model of P31-43 obtained by NMR analysis has been used to perform in silico studies to demonstrate that P31-43 is a poor ligand for DQ2 and/or T-cell receptor [69]. Altogether, these findings support the hypothesis that structural features of P31-43 may be important in the induction of different biological effects and could also have a role in the mechanism of entry into the cells.

### 6.2. Entrance of P31-43 Into the Cells: a Role for TG2? 

Several biological/toxic effects of P31-43 are very rapid and some of them may be induced sudden the peptide comes in contact with cell surface. What happens after this contact and how P31-43 enters cells are still debated issues. Experiments performed to elucidate these topics often compare the behavior of P31-43 and of peptide 57-68 (P57-68), the most immunogenic known peptide. Even if these two peptides are of similar length and composition (sequence of P31-43, LGQQQPFPPQQPY and sequence of P57-68, QLQPFPQPQLPY) they are handled by cells in a very different way. In Caco-2 cells, P31-43 is internalized by endocytosis, mainly clathrin-dependent, as methyl-β-cyclodextrin reduces its entrance. P31-43 uptake is unaffected by filipin, indicating that lipid raft/caveolae-mediated endocytosis is not involved [56,70]. On the other hand, P57-68 enters the cells mainly by a lipid raft/caveolae-mediated mechanism. After endocytosis, P31–43 is segregated in early endosomes, whereas P57-68 is transported to late endosomes [71,72]. Other studies demonstrated that P31-43 is translocated to the basal side of a Caco-2 monolayer culture [70]. In addition, P31-43 transcytosis seems to be enhanced in the presence of anti-gliadin antibodies bound to the transferrin receptor [73]. Some works have highlighted that TG2 could have a role in P31-43 uptake. The interaction of anti-TG2 antibodies with cell surface TG2 on Caco-2 cells reduces P31-43 uptake, but is ineffective on P57-68 uptake [56]. TG2 activity seems not to be involved in deranging P31-43 uptake, as enzymatic inhibitors (the competitive substrate mono-dansyl-cadaverine (MDC) and the inhibitor cystamine) do not reduce P31-43 internalization [56]. Antibodies to TG2 negatively affect also the EGF uptake by Caco-2 cells, suggesting a general role for surface TG2 in endocytosis [56]. However, P31-43 enters the HEK 293 cells, which do not express TG2, indicating that TG2 is a regulator of the peptide uptake, but its presence is not necessary for the internalization [74]. The existence of a possible surface receptor of P31-43 has been recently investigated, however no membrane protein, TG2 included, has been identified as a potential P31-43 receptor/carrier [74], leading to hypothesize that a possible mechanism of entrance into the cell can involve a direct interaction with membranes. This hypothesis is supported by the observation that the entrance into Caco-2 cells of the fluorescent-labeled P31-43 is not reduced by increasing concentrations of the unlabeled peptide [74]. In line with this finding, another study demonstrated that P31-43, but not the immunogenic P57-68, well interacts with a membrane-mimetic environment [75]. Moreover, P31-43 seems to compete with a regulator of endocytosis (the hepatocyte growth factor-regulated tyrosine kinase substrate, briefly Hrs) present on the cytosolic side of endosomes [71]; this finding suggests that P31-43 is able to directly penetrate the cell membrane or escape from endocytic vesicles. All these observations have led to suppose that P31-43 could represent the prototype of a new class of cell-penetrating peptides (CPPs) [74], short peptides able to enter cells without a necessary recognition by a receptor, that can use endocytic pathways and next escape from vesicles [76]. Finally, the ability to form foldamers, i.e., oligomers with a well-defined compact conformation, and the ability to adopt a polyproline II helical secondary structure have been described for some families of CPPs [77,78]. The description of oligomers of P31-43 and of a polyproline II conformation for this peptide are further elements supporting the idea that P31-43 could be a peptide with peculiar cell penetrating abilities. Finally, another point of reflection is that the interaction of CPPs with cell surface glycosaminoglycans, especially heparan sulphate proteoglycans, is thought as a crucial step in CPPs uptake [79]. However, no information is actually available about a link between P31-43 and proteoglycans. Interestingly, heparan sulphate proteoglycans, in particular syndecan-4, are cell surface interactors of TG2, potentially modulating its pathophysiological functions [80]. 

In this scenario, how can anti-TG2 antibodies reduce P31-43 uptake by cells? Maybe antibodies regulate the endocytosis of TG2 itself and of other TG2 membrane partners; P31-43 could merely have undergone a reduced uptake as a consequence of its interaction with membranes near TG2. Another explanation could be that antibodies trigger a signaling that at the end is able to partially block the uptake of the peptide. Further investigations are needed to clarify these issues. In any case, by reducing the uptake of P31-43, anti-TG2 antibodies also attenuate P31-43-induced effects. This observation suggests that P31-43 needs to be internalized by cells to exert its effects and that a protective role towards negative effects induced by P31-43 could be attributed to the auto-immune response to TG2. However, as described in one of the next paragraph, in CD cells this protective role of auto-antibodies seems to fail.

### 6.3. Biological Activity of P31-43

One of the first demonstrations that P31-43 is the main actor in inducing an innate immune response has been reported in the work of Maiuri et al. in 2003 [81]. They found that P31-43 induced the expression of IL15 and the activation of p38 mitogen-activated protein kinase (MAPK) as well as an increase of the apoptotic rate in cells of duodenal biopsies of CD patients but not of control patients [81]. Moreover, P31-43 enabled immunodominant epitopes to induce T-cell activation, clearly indicating that the adaptive response can be favored by an innate immune response and that both the responses can be elicited by exposure to gliadin peptides [81]. In 2007, Barone et al. demonstrated that P31-43 induced actin rearrangements and cell proliferation in a wide range of cell types, mimicking the effect of EGF [82]. They showed that P31-43 increased the level of phosphorylated EGF-receptor and ERK in Caco-2 cells, due to the persistence of EGF in endocytic vesicles. Cooperation between EGF and IL15 appeared relevant to regulate crypt enterocytes proliferation [83]. It has also been demonstrated that P31-43 is able to induce an intracellular signaling by mobilizing Ca^2+^ ions from intracellular stores and to elicit an endoplasmic reticulum (ER)-stress response in Caco-2 cells [84]. Finally, P31-43 displayed the ability to promote the migration of human dendritic cells from healthy donors with concomitant activation of p38 MAPK, important for the cytoskeletal remodeling at the basis of dendritic cells mobilization [85].

It is important to underline that several effects due to P31-43 treatment were observed in enterocytes of duodenal biopsies or other cells from CD subjects but not from control subjects [71,81,82]. From these observations, the attention of researchers has been focused to constitutive, gluten-independent, alterations in CD cells, both at the level of the intestinal mucosa and far from the main site of inflammation [86]. In CD patients, it has been possible to observe an altered intestinal permeability that persists in patients on a gluten-free diet; moreover, in CD cells, an altered cell shape and actin organization, an increased proliferation rate and signaling activation, an augmented expression of markers of cellular stress can be observed [2]. Interestingly, a celiac cellular phenotype can be partially reproduced in cells from control subjects exposed to treatments with P31-43, which cause structural alterations and activation of cell signaling and stress [2]. From all these studies emerges that CD cells, possessing constitutive altered pathways, are more susceptible to damaging effects induced by P31-43 [87].

## 7. Interplay between TG2 and 31-43

It has assumed that TG2 participates to CD onset by enhancing gluten immunogenicity, through the deamidation of specific glutamine residues, and causes the auto-immune response toward itself and also towards other self proteins, through its transamidating activity. A less defined role of TG2 in CD has emerging in correlation with the interplay between TG2 and P31-43. In general, P31-43 seems to modulate TG2 activity and expression, even if with important differences in celiac and non-celiac cells; on the other hand, inhibitors of TG2 activity can attenuate some effects induced by P31-43; finally, in some cases, P31-43 has been described as a TG2 substrate.

### 7.1. Modulation of TG2 Activity and Expression by P31-43

Regarding the influence of P31-43 on TG2, it has been demonstrated that in CD normalized duodenal biopsies (but not in non-celiac specimens) P31-43 causes an up-regulation of TG2 activity and expression [46]. A possible mechanism for TG2 up-regulation has been proposed by Luciani et al. [88]: an increase in the production of reactive oxygen species after a prolonged treatment with P31-43 could enhance TG2 level through an inhibition of TG2-ubiquitination and subsequent proteasomal degradation. As a consequence of TG2 induction, PPAR-γ, a negative inflammatory modulator, may be crosslinked by TG2 and its function may be inhibited, thus leading to an inflammatory response [88]. Additionally, in this case, the effect of P31-43 is evident only in intestinal CD cells or in “gliadin-sensitive” intestinal cell lines, i.e., Caco-2 and T84 cells [88]. In Caco-2 cells, brief treatments (30 min) with P31-43 induced TG2 activation as a consequence of Ca^2+^ mobilization from intracellular deposits [84]. Prolonged treatments also up-regulated TG2 expression [84]. A recent work has highlighted differences regarding TG2 subcellular distribution and induction by P31-43 in CD and control cells [89]. TG2 appears more associated with the cell membrane surface and with early endosomal and autophagic compartments in skin-derived fibroblasts from CD patients than in the same cells from healthy controls [89]. Moreover, P31-43 is less efficient in inducing intracellular TG2 activation in CD cells than in control ones, whereas TG2 level up-regulation is observed only in CD derived fibroblasts [89]. These differences in TG2 cellular localization and response to P31-43 in cells far from main site of inflammation can be considered an aspect of the celiac cellular phenotype, but the biological significance of such differences is still uncertain.

### 7.2. Modulation of P31-43 Effects by TG2 Inhibition

Not only P31-43 upregulates TG2 in CD cells, but also TG2 itself seems to affect the biological activity of P31-43. In works investigating how TG2 modulates P31-43 effects (or more in general the effects of the gliadin peptic-tryptic digest (PT-gliadin), enzymatic inhibitors of TG2 have often been employed (Table 1). 

Competitive aminic TG2 substrates (for example, MDC) or non specific inhibitors that binds to the TG2 catalytic site, such as cystamine, have often been used [90,91]. In addition, the availability of site direct TG2 inhibitors, as the non-peptidyl 2 2-[(2-oxopropyl)thio] imidazolium derivates, both membrane-permeable (R283) and membrane-impermeable (R281), or the peptidic 6-diazo-5-oxo-norleucine (DON)-based compounds, have enhanced the specificity of responses [91]. In some cases, antibodies to TG2 (overall the commercially available clone CUB 7402) have been used as “blocking” agents towards TG2 activity on cell surface (Table 1). However, anti-TG2 antibodies generally exert an incomplete inhibitory effect on TG2 activity, in particular on the activity of membrane-associated TG2 [92,93,94] and in some experimental conditions they seem to increase TG2 activity [3,95]. It is presumable that anti-TG2 antibodies could modulate P31-43-induced effects mainly interfering with non-catalytic functions of surface TG2 and also by reducing P31-43 uptake by cells.

In CD duodenal biopsies, the use of the TG2 inhibitor R283 reduced the T-cells activation induced by the treatment with P31-43, followed by the sequential treatment with P57-68, whereas the clone CUB 7402 exerted a very low protective effect [46]. Similarly, in a co-culture model, consisting of gliadin-sensitive T84 cells and mononuclear cells isolated from the peripheral blood of celiac patients, the apical exposition of intestinal cells to P31-43 and sequentially to P57-68 resulted in increased levels of INF-γ and other inflammatory mediators in the basal supernatants [96]. Authors demonstrated that pre-incubation of T84 cells with the clone CUB7402 prevented the ability of P57-68 to increase the level of INF-γ. Otherwise, by exposing intestinal cells to the deamidated P57-68, CUB7402 did not have any effects in reducing the increase of INF-γ level [96]. In the work of Rauhavirta et al. [97] two TG2 inhibitors, R281 and R283 were employed to verify whether the inhibition of TG2 activity influenced some effects induced by PT-gliadin, such as transepithelial resistance, cytoskeletal rearrangement, junction proteins expression and ERK phosphorylation. Authors found that both TG2 inhibitors displayed a protective action against gliadin-induced “toxic” effects in Caco-2 cells and that in some tests the cell-impermeable R281 seemed slightly more potent [97]. 

In recent work, TG2 inhibitors have been used to evaluate whether agglutination of K562 cells induced by PT-gliadin was related to the activity of cell surface TG2 [98]. Authors showed that both R281 and R283 were unable to reduce PT-gliadin-induced cells agglutination, thus demonstrating that TG2 activation on the cell surface was not essential for cellular agglutination. They also observed that P31-43, which was able to reduce the effect of PT-gliadin on cell aggregation in a medium without Ca^2+^ ions, was able to activate cell surface TG2, probably present in a closed conformation. As a consequence, the hypothesis of a direct interaction between P31-43 and cell surface TG2 was proposed as a way to induce a conformational change able to activate the enzyme [98]. In the same cell model, it has been demonstrated that CUB7402 reduced the rate of agglutination induced by PT-gliadin [99], thus supporting the idea that TG2 on the cell surface may modulate effects of PT-gliadin (and peptides encompassing P31-43 inside PT-gliadin) in a catalytic independent manner. 

A role for P31-43 and TG2 has also been proposed in the context of neurological manifestations of CD, in particular epilepsy [100]. In mice organotypic hippocampal slices, an in vitro model of epilepsy, P31-43 increased the neurotoxicity induced by kainate. A slight increased expression of both TG2 and type 6 TG was induced by exposure to P31-43 in this model; moreover, the TG inhibitor Z-DON attenuated damages induced by kainite plus P31-43.

Finally, in a very recent in vivo model, consisting of transgenic DQ8 mice over-expressing IL15 [101], an elegant demonstration of the role of TG2 in mucosal damage has been given: TG2 appeared over-expressed in the small intestine and the administration of TG2 inhibitors based on a scaffold of 3-bromo-4,5-dihydroisoxazole (ERW1041E and CK805) [102], during a gluten containing diet, prevented the development of villous atrophy. With their work authors confirmed the central role of TG2 in CD development and support the idea that TG2 may represent an important therapeutic target for CD treatment.

Thus, the use of TG2 sensible and specific inhibitors represents the best tool to investigate TG2 role in mediating the effects of P31-43, or more in general of PT-gliadin, particularly if these compounds are able to inhibit specifically extracellular or intracellular TG2.

### 7.3. P31-43 as TG2 Substrate

P31-43 appears as a good TG2 substrate in both in vitro and in situ transamidating reactions [43,46,74] even if there is no evidence that P31-43 is deamidated by TG2. In recent work, P31-43 has been found in a trimolecular covalent complex with TG2 and the cystic fibrosis transmembrane conductance regulator (CFTR) in Caco-2 cells [104]. Interestingly, P31-43 was able to inhibit the ATPase activity of CFTR on the cell surface, mimicking the effect of the presence of a defective CFTR, which is able to cause a reactive oxygen species-mediated TG2 activation and consequent autophagy impairment. The formation of the trimolecular complex was prevented when TG2 activity was suppressed by the specific membrane-permeable inhibitor Z-DON or the Ca^2+^ intracellular chelator BAPTA-AM [104]. Given the presence of very few findings about biological significance of the occurrence of P31-43 as TG2 substrate inside or outside the cell, it is clear that this issue needs more in-depth investigation.

## 8. Interplay between Anti-TG2 Antibodies and P31-43 

The idea of a connection between anti-TG2 antibodies and P31-43 in CD has an old origin. In their work, Picarelli et al. (1999) demonstrated that the exposition to P31-43 was sufficient to induce an auto-immune response: in duodenal biopsies of patients on a gluten-free diet, challenged with P31-43, an accumulation of anti-endomysial antibodies (EMA) was detected, whereas no EMA were observed in cultures of control specimens [105]. More recently, a great attention has been focused on the consequences, into the organism, of such an auto-immune response. As described above, a lot of biological effects on anti-TG2 antibodies have been described both in in vitro and in vivo systems [3]. Nevertheless, a debated issue is whether or not antibodies to TG2 can contribute to trigger CD. Interestingly, some effects exerted by anti-TG2 antibodies are identical or similar to the effects promoted by P31-43. Similarly to P31-43, antibodies induce actin rearrangement, cell proliferation and ERK phosphorylation, as well as reduce EGF endocytosis in some cell models [56,82,87,94]. Antibodies and P31-43 also increase proliferation of crypt enterocytes of CD biopsies but not of control ones [82,94]. Moreover, antibodies to TG2 mobilize Ca^2+^ ions from the same deposits engaged by P31-43, i.e., ER and mitochondria, and consequently both antibodies and P31-43 activate the intracellular TG2 [84,89]. Finally, protein phosphorylation is modulated in cells both by anti-TG2 antibodies [106] and by P31-43 [82,107]. Affinity purified antibodies against TG2 are able to increase permeability of T84 cell monolayers; similarly, P31-43 increases permeability of animal intestinal fragments [62,63]. When apoptosis has been investigated, it has been found that P31-43 induces an increase of apoptotic rate both in cell lines and in CD enterocytes, but not in control enterocytes [82], whereas anti-TG2 antibodies do not affect apoptosis in enterocytes [94]. However, anti-TG2 antibodies are able to induce apoptosis in a neuroblastoma cell line [108]. For a direct comparison, effects of both anti-TG2 antibodies and P31-43, investigated in ex vivo intestinal bioptic cultures or primary fibroblasts from control or CD patients, and also investigated in gliadin-sensitive enterocyte-like cells (Caco-2 and T84 cell lines) have been listed in Table 2.

**Figure 1:**
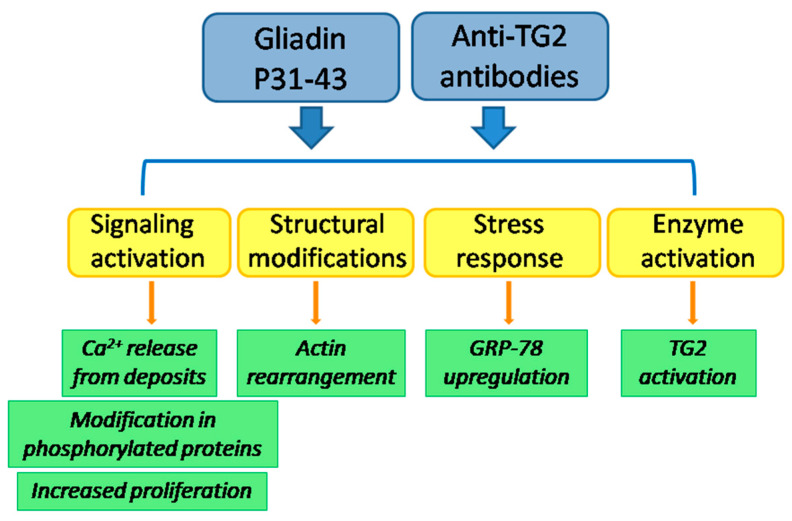
Hypothetical additive or synergic effects of antibodies to TG2 and P31-43 in the environment of CD mucosa.

Which are the biological consequences into the cell of the simultaneous presence of anti-TG2 antibodies and P31-43 have still been poorly investigated. When Caco-2 cells were treated with both antibodies to TG2 and P31-43, no additive effect was observed in cell proliferation [56]. However, by using a low amount of TG2 antibodies (not sufficient to induce the entry into S-phase), a reduction of P31-43-induced proliferation was observed. This effect was probably due to a reduction of P31-43 entry in the presence of anti-TG2 antibodies, instead to a modulation of TG2 activity on the cell surface. Such a reduction of P31-43 entry into cells has also been observed in primary cultures of skin fibroblasts from healthy subjects. Surprisingly, in CD skin fibroblasts, antibodies were unable to reduce P31-43 uptake [109]. These findings delineate a possible role of anti-TG2 antibodies in contributing to CD onset and progression, as they act on the same pathways of P31-43, with a potential additive or synergic effect (Figure 1). Antibodies also could be unable to protect intestinal cells from the entrance of P31-43, further possibly exacerbating the damage to the celiac mucosa.

Finally, it has to be considered that anti-TG2 antibodies reach all tissues and organs by blood circulation, thus potentially participating in CD extra-intestinal manifestations [3,110]. Interestingly, P31-43 has been found in urines of CD patients, thus indicating that this peptide can cross endothelial vasculature and reach the kidney for excretion [111]. Actually, there are no data about the possible consequences of the presence of P31-43 (alone or together with auto-antibodies) on the physiology of organs other than the intestine.

## 9. Conclusions and Future Perspectives

It has been well established that TG2 has two main roles in CD onset. First, TG2 enhances the adaptive immune response to gluten, through the reaction of deamidation of specific glutamine residues. Second, the transamidating activity of TG2 causes the generation of a strong auto-immune response to TG2 itself and to other self proteins. Other emerging roles could be related to the different subcellular distribution of TG2 in cells from CD or control subjects and to the influence that P31-43 could have on TG2 function and viceversa, in the context of CD. In addition, anti-TG2 auto-antibodies have the potential to influence the biology of CD cells by interacting with cell surface TG2. Antibodies seem to act on the same pathways engaged by P31-43, thus, antibodies and P31-43 could produce a synergic negative effect in celiac mucosa. In the future, investigations on CD-derived samples (such as biopsy cultures or isolated cells) with the concomitant presence of anti-TG2 antibodies and P31-43 could contribute to clarifying the role of the interplay between these three important “players”, i.e., TG2, anti-TG2 antibodies and P31-43, in CD trigger and progression.

## Figures and Tables

**Table 1:** Influence of TG2 inhibitors and anti-TG2 antibodies on the biological responses induced by P31-43 and on P31-43 uptake.

Biological Process Investigated	In Vitro Model Employed	TG2 Inhibitor or Antibody Employed	Effects Observed	TG2 Activity Involvement?
P31-43-induced proliferation	Caco-2 cells	MDC/cystamine [56] R281(unpublished data)	Inhibitors have no effect on P31-43 induced proliferation	TG2 activity seems not to be involved
		Anti-TG2 antibody CUB7402 [56]	Reduction of proliferation at low doses of antibody	TG2 activity seems not to be involved; antibodies reduce P31-43 uptake
P57-68-T cell activation after the treatment with P31-43	CD intestinal biopsies	R283 [46]	Reduction of T cell activation	TG2 activity appears involved
P57-68 -induced INF-γ production after the treatment with P31-43	T84 cells co-culture	Anti-TG2 antibody CUB7402 [96]	CUB7402 prevents INF-γ production	Maybe CUB7402 is reducing P31-43 uptake
PPAR-γ down-regulation induced by P31-43	CD (but not control) intestinal biopsies	Cystamine [92]	Reduction of PPAR-γ down-regulation	TG2 activity appears involved
Increased kainate neurotoxicity induced by P31-43	Mice hippocampal slides	Z-DON [100]	Reduction of kainate cytotoxicity	TG2 activity appears involved
P31-43-induced apoptosis	CD intestinal (but not control) intestinal biopsies and T84 cells	R283 and CUB7402 [46]	No effect on apoptosis	TG2 activity seems not to be involved
P31-43 endocytosis	Caco-2 cells	MDC/cystamine [56]	No effect on P31-43 endocytosis	TG2 activity seems not to be involved
P31-43 transcytosis	Caco-2 cells	R281 [103]	No effect on P31-43 transcytosis	TG2 activity seems not to be involved

**Table 2:** Comparison between biological effects induced by anti-TG2 antibodies and the same/similar effects induced by P31-43 in control cells/biopsy cultures, in CD cells/biopsy cultures and in gliadin-sensitive cells (Caco-2 and T84 cells). N.d. not determined.

Biological Process Investigated	Induced by Antibodies			Induced by P31-43		
	In Control Cells/Biopsies	In CD Cells/Biopsies	In Gliadin Sensitive Cells	In Control Cells	In CD Cells	In Gliadin Sensitive Cells
**Cell proliferation**	No effect [94]	Increased [94]	Increased [56]	No effect [82]	Increased [82]	Increased [82]
**ERK activation**	N.d.	N.d.	Increased [56]	N.d.	N.d.	Increased [82]
**Ca^2+^ ions mobilization**	N.d.	N.d.	Increased [57]	N.d.	N.d.	Increased [84]
**Protein phosphorylation**	N.d.	N.d.	Several proteins modulated [106]	Increased phosphory-lation of paxillin and Fak [107]. No tyrosine phosphory-lation [46]	Increased phosphory-lation of paxillin and Fak, more than in control cells [107]. Increased tyrosine phosphory- lation [46]	Increased EGF-receptor phosphory-lation [82]
**GRP-78 expression**	N.d.	N.d.	Increased (unpublished data)	N.d.	N.d.	Increased [84]
**Actin rearrangement**	N.d.	N.d.	Increased [56]	Increased in fibroblasts, not increased in duodenal enterocytes [46]	Increased in fibroblasts [107] and in duodenal enterocytes [46]	Increased [46,82]
**Intracellular TG2 activity**	N.d.	N.d.	Increased [57]	Increased [89]	Increased (less than in control cells) [89]	Increased [84]
**Apoptosis**	No effect [94]	No effect [94]	N.d.	No effect [81,82]	Increased [81,82]	Increased [46]
**Vesicular trafficking**	N.d.	N.d.	Interference with EGF endocytosis [56]	Transient delay of EGF/EGF-receptor trafficking [87]	Prolonged delay of EGF/EGF-receptor trafficking [87]	Interference with EGF endocytosis [82]

## Abbreviations

TG2	type 2 transglutaminase
TG	transglutaminase
CD	celiac disease
ECM P31-43	extra-cellular matrix α-gliadin peptide 31-43
P57-68	α-gliadin peptide 57-68
HLA	human leukocytes antigen
APC	antigen presenting cell
IL15	interleukin 15
MDC	mono-dansyl-cadaverine
CPPs	cell-penetrating peptides
EGF	epidermal growth factor
PT-gliadin	gliadin peptic-tryptic digest
DON	6-diazo-5-oxo-norleucine
INF	interferon
CFTR	cystic fibrosis transmembrane conductance regulator
